# COVID-19 and Placental Infection: Are Fetal Survivors at Risk of Long-Term Cardiovascular Complications?

**DOI:** 10.7759/cureus.38077

**Published:** 2023-04-24

**Authors:** Jordyn Mullins, Dominic J Bewley, Angelica Oviedo

**Affiliations:** 1 Department of Physiology and Pathology, Burrell College of Osteopathic Medicine, Las Cruces, USA

**Keywords:** pregnancy, fetal hypoxia, heart disease, intervillositis, placenta, coronavirus, sars-cov-2

## Abstract

As we enter the fourth year of the coronavirus disease 2019 (COVID-19) pandemic, it has become obvious that adult survivors of severe acute respiratory syndrome coronavirus 2 (SARS-CoV-2) infection are susceptible to numerous complications in various organ systems. SARS-CoV-2 placental infection is an unanticipated complication of COVID-19 during pregnancy. We hypothesize that fetal survivors of SARS-CoV-2 placentitis are susceptible to long-term cardiovascular complications.

## Editorial

Background

As we recently passed the three-year mark with the pandemic, we are beginning to see the effects that coronavirus disease 2019 (COVID-19) has caused on a global spectrum [1]. To fully understand the long-term effects will take decades. There was an increase in maternal death rates attributed to COVID-19 as compared to pre-pandemic death rates [2]. In addition, there was an increased requirement for intensive care treatment, including extracorporeal membrane oxygenation (ECMO), in severe acute respiratory syndrome coronavirus (SARS-CoV-2) infected mothers [2,3]. In addition, it is clear that COVID-19 can lead to stillbirth [4]. Recent studies have shown that fetal demise is typically due to SARS-CoV-2 binding to the angiotensin-converting enzyme 2 (ACE2) receptors of the trophoblastic cells of the placenta, causing intervillositis and perivillous fibrin deposition, which causes a severe hypoxic environment for the fetus, resulting in death [5]. The most common mechanism of fetal death appears to be severe hypoxia resulting from placental infection and not direct SARS-CoV-2 viral infection of the fetus [4]. Increased ACE2 receptor expression in pregnant women likely contributes to more severe maternal illness [6]. However, it is unclear whether there are adverse effects seen in surviving fetuses. Perhaps fetal survivors may have an increased risk of cardiovascular diseases, such as hypertension, atherosclerosis, and myocardial disease, in later life. In addition, there may be “silent hypoxia” in COVID-19-infected mothers that may affect the fetus [6]; this is characterized by low blood oxygen as measured by a pulse oximeter without overt clinical symptoms in the mother. This is an additional hypoxic insult that may affect the placenta and fetus, on top of the changes due to direct SARS-CoV-2 placental infection. In addition, it is unclear whether additional factors, such as neonatal patent ductus arteriosus and gestational age at the time of infection, play a role in long-term disease processes in survivors of SARS-CoV-2-infected pregnancies.

Hypothesis

The effects of the SARS-CoV-2-infected placenta will be detrimental to the surviving fetus later in life. Intrauterine exposure to maternal disease processes, such as cyanotic heart disease, heart failure, and pulmonary hypertension, has been shown to affect the future cardiovascular health of the fetus. Intrauterine asphyxia is also well known to have long-term effects on the fetus. We propose that SARS-CoV-2 infection of the placenta via the mother and the maternal effects of COVID-19 have similar long-term effects on the surviving fetus. The severity of the placental infection likely will also play a role.

Support for the hypothesis

Fetuses who survive intrauterine chronic fetal hypoxia or acute fetal hypoxia have an increased risk of detrimental effects that may manifest in the pediatric or adult time period. We propose that survivors of SARS-CoV-2-infected pregnancies are likely to have long-term effects in adulthood.

Chronic Fetal Hypoxia

Chronic fetal hypoxia resulting from maternal conditions has been shown to have effects on the neonate such as poor growth [7]. In known chronic fetal hypoxic conditions resulting from maternal cardiovascular diseases, such as cyanotic heart disease, heart failure, or pulmonary hypertension, the fetus may have time to compensate for changes in cardiovascular function as a protective mechanism. In addition, fetal hemoglobin also allows the fetus to compensate for maternal hypoxia due to the markedly higher affinity for oxygen as compared to adult hemoglobin. However, SARS-CoV-2 placental infection may occur too rapidly for the fetus to compensate for cardiovascular alterations [7].

Infants who survive maternal chronic hypoxic disease during pregnancy have been shown to be growth-restricted, which is considered a risk factor for cardiovascular disease. In adulthood, these surviving fetuses have an increased risk of arterial hypertension, increased systolic blood pressure, premature coronary heart disease, stroke, and diabetes. There have been similar findings in animal models of chronic intrauterine hypoxia [7].

The mechanism of these long-term cardiovascular changes due to intrauterine hypoxia is likely epigenetic changes in myocardial and vascular development. Prenatal programming likely leads to long-term changes in the expression of numerous factors (heat shock proteins, beta-adrenergic receptors, endothelial nitric oxide synthase), which are responsible for the regulation of cardiac and vascular function [7].

In addition, cardiac myocyte response to chronic intrauterine hypoxia, such as apoptosis or exiting the cell cycle, likely contributes to fewer but hypertrophied cardiac myocytes. Although this is more energy efficient in the fetus, it likely results in less compliant ventricles, which lead to cardiovascular disease in adulthood [7].

Acute Fetal Hypoxia

Severe acute hypoxia is well known to have detrimental effects on the fetus. Adaptive cardiovascular responses aim to preserve blood flow to the brain and heart at the expense of other organs. In a sheep animal model of acute intrauterine asphyxia, it has been shown that the surviving fetuses can have brain injury due to disruption of blood flow. The mechanisms also include oxidative stress responses that lead to deficiencies in nitric oxide pathways which may damage the cardiovascular system. Despite advances in care, survivors of acute intrauterine asphyxia frequently have significant brain injury (hypoxic ischemic encephalopathy) [8].

Hypoxia and acidosis from acute intrauterine asphyxia contribute to postnatal pulmonary hypertension. In the developing fetus, the right ventricle is dominant and responsible for moving blood into the high-pressure pulmonary artery. In fetuses exposed to hypoxic environments, the right ventricle is at risk of damage; cardiac dysfunction and pulmonary hypertension would be expected to occur postnatally. This can then lead to poor pulmonary blood flow, ventilation-perfusion mismatching, decreased preload of the systemic circulation, and the resulting reduction in cardiac output. These cardiovascular changes also contribute to the development of hypoxic-ischemic encephalopathy and may ultimately result in hemodynamic instability [8].

Effects of Chronic and Acute Hypoxia on Transitional Circulation

The cardiovascular conditions mentioned above may result in fetal growth restriction (FGR), which is generally defined as a birth weight less than the 10th percentile when controlled for gestation and sex [8]. Changes in fetal programming due to hypoxic conditions lead to alterations in physiology, metabolism, and structural components of the heart, which can then result in FGR. This condition may be associated with several causes. However, it is most often caused by placental insufficiency resulting in an inadequate supply of oxygen and nutrients for development.

It appears that FGR is not common in SARS-CoV-2-infected mothers. In 2022, Wilkinson et al. conducted an analysis of over 36,000 births and did not find significant data that would confirm that FGR is a common result of maternal infection [9]. However, the underlying vascular compensation mechanisms involved in usual cases of FGR may play a role in SARS-CoV-2-infected placentas. When a fetus is delivered, it undergoes complex physiologic adaptations while it transitions from obtaining oxygen from the placenta (fetal circulation) to obtaining oxygen from the lungs (neonatal circulation). Both FGR and SARS-CoV-2-infected placentas likely have detrimental effects on the physiology of transitional circulation that takes place upon delivery since the hypoxia common to both can cause hemodynamic instability [8]. FGR serves as a useful surrogate for fetal effects due to SARS-CoV-2 placentitis.

Animal models of acute and chronic fetal hypoxia on transitional circulation have helped elucidate the mechanisms that likely lead to long-term detrimental effects in survivors. These changes involve persistent changes in cardiac function and vascular remodeling [8]. Studies on human FGR cohorts across different ages have shown higher systolic, diastolic, and mean blood pressure (BP) [10]. They also show arterial changes, including higher resistance, wall stiffness, and impedance when compared to appropriate for gestational age (AGA) infants [10]. These changes persist into childhood and adulthood and can manifest clinically as increased BP [11,12].

Pathophysiologic Mechanism of Long-Term Cardiovascular Changes in Fetal Hypoxia

Fetal distress has been reported in nearly 50% of all births in mothers infected with SARS (CoV-1 or CoV-2) [13]. In other similar disease states, hypoxia in the placenta has been shown to affect fetal vascular development. This is likely mediated by α-klotho, a protein produced by the placenta, which has effects on fetal vasculature. Decreased α-klotho in hypoxic states causes changes that include thickening of arteries (which leads to hypertension and other cardiovascular impairments later in life), thickened pulmonary arteries, reduced pulsatility, and greater right ventricular contraction when compared to healthy fetuses at the same gestational age [8].

Patent Ductus Arteriosus

The patent ductus arteriosus (PDA) in the developing fetus allows oxygenated blood from the placenta to bypass the pulmonary circulation and enter the systemic circulation via the descending aorta. This patency is maintained by the presence of high blood flow along with circulating prostaglandins from the placenta [14]. After delivery, the increase in fetal oxygen tension that occurs results in functional closure of the ductus arteriosus and eventual anatomical closure in the first couple weeks of neonatal development.

It is abnormal to observe post-natal patency in the ductus arteriosus; this can be seen in many pathologic conditions [14]. The conditions of a SARS-CoV-2 infected placenta and resulting hypoxia could potentially also lead to a PDA. This is not known and should be investigated in the future. 

Post-natal complications of PDA can include respiratory distress syndrome and pulmonary hypertension [14]. It is possible that PDA may contribute to additional post-natal complications in some survivors of SARS-CoV-2 placentitis.

Vaccinated vs. Unvaccinated Mothers

The two-dose regimen of the COVID-19 vaccine leads to the production of immunoglobulin gamma (IgG) antibodies against the virus in the mother’s immune system. It has also been documented that through the utilization of IgG receptors in the placenta, these antibodies are able to traverse the placenta and confer up to six months of protection postpartum to the fetus/infant. One study documented that out of 379 infants hospitalized due to COVID-19 infection, 88% of them were born to unvaccinated mothers. Fifteen percent of that 88% received mechanical ventilation, vasoactive infusion, and/or ECMO, and one infant died [15]. Other studies have shown that there is no direct evidence of fetal risk from maternal vaccination during pregnancy [16]. It is therefore recommended that pregnant women receive the COVID-19 vaccine based on scientific data that shows protection with the vaccine; there are potential drastic outcomes due to lack of maternal vaccination.

Evaluation of hypothesis

To assess the effects of SARS-CoV-2 placental infection on the surviving fetus, we propose long-term monitoring throughout childhood and adulthood for these patients. This study should include an adequate number of survivors; these numbers are easily reached during the various COVID-19 surges. Evaluation should include studies of cardiac function such as echocardiogram and blood pressure measurements. 

It is important to consider the selection of controls for these studies. The time of the pandemic was extraordinarily stressful for all people, so there may be stress effects in fetuses born to non-COVID-19-infected mothers during the pandemic. Controls should be selected using gestational age and sex-matched controls who were born on the same day to non-COVID-19-affected mothers in the same hospital.

Conclusion

SARS-CoV-2 placentitis resulting from maternal infection during pregnancy likely has long-lasting effects on survivors. Prior to the pandemic, numerous studies have shown long-term cardiovascular effects in survivors of acute perinatal asphyxia and chronic intra-uterine hypoxia (infants with FGR). The underlying mechanisms that lead to long-term damage likely result from the effects of acute and chronic hypoxia on the pre-natal and peri-natal time periods. These changes are likely to affect survivors of fetal SARS-CoV-2 placentitis well into adulthood.
